# Radium in the Treatment of Cancer

**Published:** 1910-12

**Authors:** 


### Radium in the Treatment of Cancer.

We abstract from the *Revue de Chirurgie* (July 10, 1910,) that, in a recent discussion before the Paris Surgical Society, in which quite a number of members participated, it was the consensus of opinion that radiotherapy, either by the extra or intra-cancerous application, had not proven itself to be a real curative measure. It was asserted by some, however, that a purely local action on the cancerous tissue had at times been noticed, rendering previously inoperable tumors, especially of the womb, operable. Marked retrogression of malignant growths had in a few instances been produced.

Nelation claimed to have observed in a case of cancer of the cervix uteri with involvement of the anterior cul-de-sac that, following one application of radium, the parts, one month after, seemed to be free and healthy. Lucas-Championniere mentioned the case of a young woman with a very rapidly growing cancer of the vagina, deemed by him inoperable, and with impending death. Radium treatment effected a complete local healing with cicatricial formation and arrest of the disease in situ.

This same patient, nevertheless, seven months later, suffered with sciatic pains, due to a deep recurrence, to which she quickly succumbed.—*Larue, in N. O. M. and S. Journal.*

